# The generalizability of psychotherapy efficacy trials in major depressive disorder: an analysis of the influence of patient selection in efficacy trials on symptom outcome in daily practice

**DOI:** 10.1186/1471-244X-12-192

**Published:** 2012-11-08

**Authors:** Rosalind van der Lem, Wouter WH de Wever, Nic JA van der Wee, Tineke van Veen, Pim Cuijpers, Frans G Zitman

**Affiliations:** 1 Department of Psychiatry, Leiden University Medical Center/Rivierduinen, Albinusdreef 2, PO box 9600, Leiden, RC, The Netherlands; 2 Leiden Institute for Brain and Cognition, Albinusdreef 2, PO box 9600, Leiden, RC, The Netherlands; 3 Department of Clinical Psychology, VU University, Van der Boechorststraat 1, Amsterdam, BT, 1081, the Netherlands; 4 Kijvelanden/Het Dok, Zomerhofstraat 76-90, Rotterdam, CM 3032, The Netherlands

**Keywords:** Major depressive disorder, Psychotherapy efficacy trials, Exclusion criteria, Generalizability, Treatment outcome, Symptom outcome, Routine clinical practice, Routine outcome monitoring

## Abstract

**Background:**

Treatment guidelines for major depressive disorder (MDD) are based on results from randomized clinical trials, among others in psychotherapy efficacy trials. However, patients in these trials differ from routine practice patients since trials use stringent criteria for patient selection. It is unknown whether the exclusion criteria used in psychotherapy efficacy trials (PETs) influence symptom outcome in clinical practice. We first explored which exclusion criteria are used in PETs. Second, we investigated the influence of commonly used exclusion criteria on symptom outcome in routine clinical practice.

**Methods:**

We performed an extensive literature search in PubMed, PsycInfo and additional databases for PETs for MDD. From these, we identified commonly used exclusion criteria. We investigated the influence of exclusion criteria on symptom outcome by multivariate regression models in a sample of patients suffering from MDD according to the MINIplus from a routine clinical practice setting (n=598). Data on routine clinical practice patients were gathered through Routine Outcome Monitoring.

**Results:**

We selected 20 PETs and identified the following commonly used exclusion criteria: ‘a baseline severity threshold of HAM-D≤14’, ‘current or past abuse or dependence of alcohol and/or drugs’ and ‘previous use of medication or ECT’. In our routine clinical practice sample of patients suffering from MDD (n=598), presence of ‘current or past abuse of or dependence on alcohol and/or drugs’ had no significant influence on outcome.‘Meeting a baseline severity threshold of HAM-D≤14’ and ‘previous use of medication or ECT’ were associated with better outcome, but the explained variance of the models was very small (R^2^=2-11%).

**Conclusions:**

The most consistently used exclusion criteria are not a major threat to the generalizability of results found in PETs. However, PETs do somewhat improve their results by exclusion of patients with minor depression and patients who used antidepressants prior to psychotherapy.

## Background

In the development of guidelines, randomised controlled trials (RCTs) and meta-analyses thereof are considered the most reliable source of evidence. However, it is unknown to what extent the results of these RCTs are generalizable to routine clinical practice. In RCTs, much effort is put in optimising the internal validity, i.e. the possibility to determine to what extent the observed efficacy is reproducible and attributable to the investigated treatment. The internal validity of trials is improved by the use of strict criteria for patient selection. While this is very important for methodological and ethical reasons, it has been demonstrated that the use of eligibility criteria may well hamper the generalizability (external validity) of the results [1-6]. In trials of antidepressant treatment of major depression (MDD), a fairly consistent set of exclusion criteria is used [2]. Based on this set of criteria, we and others found that only 12-34% of the patients who received treatment for MDD in routine outpatient psychiatric care settings and fee-for-service private practice were eligible for participation in an antidepressant efficacy trial (AET) [1,3][7]. Some studies showed that eligible patients had a better treatment outcome than non-eligible patients in routine outpatient care [8]. In contrast, we found that only exclusion of minor depression was associated with better treatment outcome [9]. Thus, the AET exclusion criteria had a limited influence on treatment outcome.

Whereas the influence of exclusion criteria on treatment outcome is a topic in research on AETs, this is not the case for research on psychotherapy efficacy trials (PETs). To our best knowledge, only one study reported on the eligibility of ‘real life’ patients for PETs. A total of 95% of patients with several common psychiatric disorders were eligible for at least one PET and 75% for two or more [10,11]. However, the authors did not investigate the comparability of the exclusion criteria used in the PETs. Lack of consistency in this respect may diminish the unequivocality of the results of PETs and thereby the generalizability of the results to ‘real life’ patients.

In this paper, we present the effects of the most used exclusion criteria of PETs on eligibility of ‘real life’ patients. First, we identified the exclusion criteria used in PETs. Subsequently, we examined the proportion of patients with unipolar depression eligible for PETs, applying the most used exclusion criteria, to a sample of ‘real life’ patients with major depressive disorder (MDD) from the Leiden Routine Outcome Monitoring Study [12]. Finally, we investigated the influence of eligibility for PET on symptom outcome from the first treatment step, in this sample.

## Methods

### Identification of exclusion criteria in PETs

In line with previous research on the consistency in the use of exclusion criteria in AETs [2], we performed a search in PubMed and PsycInfo for publications in English on PETs for adult patients suffering from MDD. Furthermore, we checked the reference lists of the included publications for relevant studies. We also consulted: http://www.psychotherapyrcts.org. This website is composed by a group of researchers from the VU University Amsterdam, the Netherlands, and contains a database of RCTs and comparative studies of the effect of psychotherapy on adult depression. We selected PETs in which outpatient treatment was investigated and in which one of the comparison groups was treated with either only individual cognitive behavioral therapy (CBT) or individual interpersonal therapy (IPT) as these two treatments are usually incorporated in treatment guidelines. For all the studies that met our inclusion criteria, we retrieved eligibility criteria from their Methods sections.

### The Dutch mental health care system and treatment steps for MDD

The Dutch mental health care system is organized in a stepped-care-manner and uses treatment guidelines which are based on evidence from AETs and PETs. Patients with mood complaints visit their general practitioner (GP) first. GPs will refer patients with a first episode of a mild depression either to counseling sessions or prescribe antidepressants. The Dutch and many other guidelines recommend that patients with moderate depression should be treated with CBT or IPT or pharmacotherapy, based on the patient’s preferences [13-15]. Reasons to refer patients to a regional mental health provider (RMHP) are a preference of patients for psychotherapy (only provided by psychotherapists), severity or recurrence of depression, and non-response to the GP’s treatment. After baseline-assessment and a clinical interview at our RMHP, patients are offered treatment steps as recommended by the guidelines. If patients are not too severely ill and have sufficient mastery of the Dutch language, they are eligible for psychotherapy when this is their preferred treatment.

### Patients

Data on ‘real life’ patients were drawn from the Leiden Routine Outcome Monitoring Study [12]. In 2002, the RMHP Rivierduinen (service area with 1.1 million inhabitants), in collaboration with the University Medical Hospital Leiden, implemented ROM and evidence-based, stepped care protocols. In ROM, all patients referred to the RMPH for treatment of a mood, anxiety or somatoform disorder have an extensive baseline assessment. Treatment progress is then assessed at three to four monthly intervals and before starting a new treatment step. The baseline assessment comprises, besides a clinical interview, a standardized diagnostic interview (Mini-International Neuropsychiatric Interview Plus [16], the collection of sociodemographic and socioeconomic data, the administration of disease-specific severity-scales, and general measures of health. All ROM instruments are administered by independent and specially trained research nurses. For a more extensive description of ROM, we refer to the design paper [12]. Patients were between 18–65 years of age, referred for treatment between January 2002 and January 2007 to the RMHP Rivierduinen, and had at least one follow-up assessment.

Since the goal of this research was to evaluate the generalizability of the results of psychotherapy trials, which generally use symptom reduction or remission on an observer rated instrument as primary outcome, we used the data collected with equivalent instruments in our ROM system. In ROM, MDD was diagnosed with the Dutch version of the MINI-Plus and depression severity was assessed with the Montgomery Asberg Depression Rating Scale (MADRS, [17]). To explore putative selection bias, we performed a lost to follow up analysis by comparison of patients only assessed at baseline with those included in our study. We investigated the eligibility and the effects of eligibility on outcome in all MDD patients referred for treatment irrespective of the treatment they received (antidepressants or psychotherapy). Since the type of treatment that patients receive might influence outcome, we adjusted for ‘treatment modality’ in these analyses. To examine the effects of eligibility to PETs on treatment results of psychotherapy specifically, we also conducted the analyses in patients who were actually treated with CBT or IPT.

### Effects of exclusion criteria on symptom outcome in daily practice

In line with previous research on exclusion criteria in AETs [1-3,18,19], we explored the influence on outcome of exclusion criteria used in >75% of the PETs. In line with the methodology of PETs, we defined outcome in our daily practice population as the extent of improvement on the MADRS (difference between baseline and post treatment), and in line with the methodology of both AETs and PETs also as proportion of responders (50% reduction of symptoms), and as proportion of remitters (MADRS ≤10) [20] after the first step treatment for MDD.

### Statistical analysis

The effects of the exclusion criteria on outcome were computed by univariate and multivariate linear and logistic regression analyses. In the multivariate (adjusted) analyses on each individual exclusion criterion, the effects of the exclusion criterion on outcome were adjusted for age, gender and all the other exclusion criteria. In the analysis on all MDD patients we also adjusted for ‘treatment modality’ (type of treatment that the patients received: antidepressants, psychotherapy or a combination of both). For the lost to follow-up analyses, independent sample t-tests and Chi-square analyses were carried out. The statistical software package SPSS 16.0 was used.

## Results

### Identification of exclusion criteria in PETS

Our PubMed search yielded 3931 potentially relevant titles of studies. Another 203 potentially relevant studies were retrieved from reference lists of manuscripts and from the database of the VU University Amsterdam. The majority of these studies were carried out in specific subgroups, such as elderly, ethnic minorities or patients with specific somatic co morbidity (n=4085). Therefore, these studies were excluded. Another 22 manuscripts were excluded because they were duplicates between the three databases. Of the remaining 27 PETs, seven were excluded for the following reasons: in one PET the psychotherapeutic intervention appeared to include a prominent role for the spouse of the patients [21]; in another, the use of in- and exclusion criteria was mentioned but not made explicit [22] ; five PETs were excluded as they used the same datasets as other studies already part of our review [23][24][25][26][27]. Finally, 20 PETs could be included [28-42]; [43-47]. In 18 studies (90%), individual CBT was one of the intervention arms and in 5 studies (25%) individual IPT was. In 12 PETs (60%), antidepressants (most frequently tricyclic antidepressants) were used as comparison treatment. No PETs used treatment as usual or a waiting list group as control group.

From the PETs, we identified 38 exclusion criteria, which we grouped into the following 15 categories (+ number of studies that reported the use of this criterion): 1) bipolar disorder or a history of a (hypo-manic episode (19 studies); 2) history of schizophrenia or psychosis or psychotic features (18 studies); 3) current or past abuse of or dependence on alcohol and/or drugs (17 studies); 4) not meeting a minimum severity threshold (16 studies); 5) previous use of medication or electro convulsive therapy (ECT) (14 studies); 6) comorbid personality disorder (12 studies); 7) cognitive disorders (11 studies); 8) somatic concerns (11 studies); 9) receiving other treatment at the start of the trial (10 studies); 10) anxiety disorder as a primary diagnosis (9 studies); 11) contra indication for the use of medication (9 studies); 12) suicidality (8 studies); 13) previous psychotherapy (8 studies); 14) comorbid Axis I disorders (5 studies) and 15) crisis situation (4 studies). In line with the model of Zimmerman and colleagues on commonly used exclusion criteria in AETs [2], we planned to examine the criteria that were used in more than 75% of all PETs:, which were: 1) bipolar disorder or a history of a (hypo-) manic episode (95%); 2) schizophrenia, a history of psychosis or psychotic features (90%); 3) current or past abuse of or dependence on alcohol and/or drugs (85%) and 4) not meeting a minimum severity threshold (80%; most common: cut-off score of 14 on the Hamilton Rating Scale for Depression [48] HAM-D-17). ‘Previous use of medication or ECT’ was used in only 70% of the PETs, but we included this criterion in our further analyses as we hypothesized that it may have a large impact on eligibility of ‘real life’ patients. Bipolar disorder and psychosis are considered to be different entities from MDD. Not only in PETs, but also in clinical practice, patients are treated differently if they have bipolar disorder or a history of a (hypo-) manic episode, or a history of schizophrenia or psychosis or psychotic features. Therefore, these exclusion criteria are not likely to jeopardize the generalizability of the results of PETS for MDD to daily practice. Furthermore, we included the frequently used criteria ‘current or past abuse or dependence on alcohol and/or drugs’ and ‘not meeting a minimum severity threshold’ in our analyses. Comorbid substance abuse and relatively mild depression often occur in daily practice. Therefore, the frequently used exclusion criteria, ‘current or past abuse or dependence on alcohol and/or drugs’ and ‘not meeting a minimum severity threshold’ are likely to jeopardize the generalizability of the results of PETs to daily practice. Since in clinical practice alcohol abuse might be more common than drug abuse, we studied the effects of ‘current or past abuse or dependence on alcohol’ and ‘current or past abuse or dependence on drugs’ separately. Table 1, shows the exclusion criteria, the 15 summarized categories and their frequencies as identified in PETs.

**Table 1:** (Categories of) exclusion criteria found in psychotherapy efficacy trials

**Categorical exclusion criterion**	Subtypes of exclusion criteria included in Category*	**Proportion of trials using the criteria**
Bipolar disorder or history of (hypo-) manic episode		95%
Schizophrenia, a history of psychosis or psychotic features		90%
Current or past abuse or dependence on alcohol and/or drugs	- Alcohol abuse or dependence	85%
	- Drug abuse or dependence	
Not meeting a minimum severity threshold		80%
Previous use of medication or ECT	- ECT less than 6 months before start of trial	70%
	- History of use of a tricyclic antidepressant	
	- Use of amitriptyline less than 3 months prior to trial	
	- Use of imipramine less than 3 months prior to trial	
	- Use of paroxetine less than 1 year prior to trial	
	- Use of any antidepressant less than 2 months prior to trial	
	- Use of any antidepressant less than 1 month prior to trial	
	- Use of any antidepressant less than 2 weeks prior to trial	
	- Current use of an antidepressant	
Co morbid personality disorder	- Borderline personality disorder	60%
	- Antisocial personality disorder	
	- Schizotypical personality disorder	
Cognitive disorders	- Cognitive disorders in general	55
	- Organic brain syndrome	
	- Delirium or dementia	
	- Mental retardation	
Somatic concerns	- Somatic co morbidity in general	55%
	- Co morbid somatisation disorder	
Anxiety disorder as primary diagnosis	- Generalized anxiety disorder	45%
	- Specific phobia	
	- Obsessive-compulsive disorder	
	- Panic disorder	
Contra indication for the use of medication in general		45%
Suicidal ideation		40%
Previous psychotherapy, with or without success	History of psychotherapy	
	- Psychotherapy less than 5 years prior to trial	
	- Psychotherapy less than 2 years prior to trial	
	- Psychotherapy less than 1 year prior to trial	
	- Psychotherapy less than 2 months prior to trial	
	- Current psychotherapy	
Psychiatric co morbidity in general, including eating disorders		25%
Crisis	- Need for immediate intervention	20%
	- Indication for admission	

*If no subtypes are mentioned, the categorical exclusion criterion was reported in the same way in all trials.

### Patients

Between January 2002 and January 2007, 1653 outpatients seeking treatment at RMHP Rivierduinen suffered from MDD according to the MINIplus. 774 patients (46%) had at least one follow-up assessment. Extensive chart-review was done for those 774 patients. As we confined our study to patients with unipolar depression, we excluded 42 patients who were suspected to have a bipolar disorder or psychotic features. Furthermore, 132 patients had to be excluded from further follow-up analysis due to missing information on treatment, admission to an inpatient-clinic during follow-up, remission on the MADRS at baseline or a time-span between baseline and follow-up assessment which we considered either to be too short (less than four weeks) or too long (more than 52 weeks) to provide reliable information. Finally, 598 patients were selected for follow-up analysis. Of these 598 patients, 80 patients only received individual psychotherapy (CBT or IPT) for MDD; 82 patients received only antidepressants; 90 patients received psychotherapy for a comorbid disorder other than MDD or the focus of psychotherapy could not be extracted from chart review; 167 patients received a combination of psychotherapy for MDD and antidepressants; 90 patients received antidepressants and social supportive counseling; 89 patients received other forms of treatment, i.e. mood stabilizers; group therapy, training courses. Clinical and demographical characteristics of the whole sample as well as the 80 patients who received psychotherapy only are reported in Table 2. In an earlier study on this sample we examined selection bias, due to loss to follow up of patients. We showed that the patients of this sample were very similar to the patients who were lost to follow up [7]. In Table 2, we present the baseline features and symptom outcome in ROM patients suffering from MDD.

**Table 2:** Baseline features and symptom outcome in ROM patients suffering from MDD

	**All MDD patients (n=598)**	**Patients who received psychotherapy only (n=80)**
**Age (in years)**	39.3 (SD 11.3)	36.2 (SD 10.8)
**Gender (% female)**	66.7% (n= 399)	73.8% (n=59)
**MADRS pre treatment**	25.9 (SD 6.5)	24.1 (SD 6.0)
**MADRS post treatment**	18.2 (SD 9.4)	16.5 (SD 9.1)
**Treatment outcome**		
Within-Group Effectsize^1^	1.16	1.28
Proportion of responders^2^	29.1%	35.0%
Proportion of remitters^3^	22.6%	27.5%
**Ethnicity**		
Netherlands	84.8%	76.4%
Turkey/Morocco	5.1%	5.5%
Suriname/Antilles	3.1%	4.2%
Other	7.0%	13.9%
**Marital Status**		
Married/Cohabitating	52.8%	44.4%
Divorced/Widowed	16.6%	22.2%
Single/LAT	30.0%	33.3%
**Employment Status**		
Employed	34.3%	34.7%
Not Employed	26.1%	34.7%
Sickness Benefit	39.0%	30.6%
Retired	0.6%	0%
**Educational level**		
Low	12.3%	9.7%
Intermediate low	33.1%	23.6%
Intermediate high	38.4%	40.3%
High	16.2%	26.4%

Caption:

^1^ Within Group Effectsize is a definition of treatment outcome often used in PETs and defined as: the extent of improvement (Δ MADRS pre- and post treatment within the group under investigation) adjusted for the standard deviation pre treatment.

^2^ Response is defined as a 50% reduction of symptoms on the MADRS.

^3^ Remission is defined as MADRS≤10.

### Effects of exclusion criteria on symptom outcome

As we confined our study to unipolar depression, we excluded patients with a ‘bipolar disorder or a history of a (hypo-) manic episode’ and patients with a ‘history of schizophrenia or psychosis or psychotic features’ from our daily practice sample. Hence, we did not explore the effects of these two frequently used exclusion criteria in PETs. We did analyze the effects of the exclusion criteria ‘current or past abuse or dependence on alcohol and/or drugs’, ‘not meeting a minimum severity threshold’ and ‘previous use of medication or ECT’ on outcome.

In the literature, the baseline severity threshold (a cut-off score of 14 on the HAM-D-17 for PETs) is usually defined as a score on the HAM-D-17. In our routine clinical practice (ROM), depression severity is assessed with the MADRS. To enable comparison, we converted the scores MADRS of the ROM patients into HAM-D-17 scores with the equation proposed by Zimmerman [49]: MADRS = 1.43 X HAM-D + 0.87. Recently, the Item Response Theory (IRT) was suggested to be a more reliable method to convert MADRS scores into HRSD17 scores. As a sensitivity analysis, we also used the IRT method [50] procedures yielded similar results for the conversion of the MADRS scores into HAM-D-17 scores.

Table 3 shows the proportions of patients meeting the exclusion criteria for all 598 patients with MDD, as well as for the 80 patients treated with psychotherapy. In the group of all MDD patients, the criterion ‘Previous use of medication or ECT’ had the largest effect on proportion of eligible patients. In the 80 psychotherapy patients, the criterion ‘not meeting baseline severity threshold’ had the strongest effect.

**Table 3:** Exclusion criteria in ROM patients suffering from MDD

**Exclusion criterion**	**All MDD patients (n=598)**	**Patients who received psychotherapy only (n=80)**
**Current or past abuse or dependence of drugs**	2.3% (n=14)	5.0% (n=4)
**Current or past abuse or dependence of alcohol**	5.0% (n=30)	2.5% (n=2)
**Not Meeting Baseline Severity Threshold**	21.9% (n=131)	30.8% (n=24)
**Previous use of medication or ECT**	44.1% (n=230)	13.8% (n=11)
**(all patients received antidepressants, none of the patients received ECT prior to psychotherapy**	Missing data: n=77	Missing data: n=0

Table 4 shows the joint effects of the exclusion criteria on symptom outcome. In the group of all 598 depressed unipolar patients the criterion ‘current or past abuse of or dependence on alcohol and/or drugs’ had no significant influence. In the 80 psychotherapy patients, patients that met this criterion were too few in number for analysis of the effect. In the group of all 598 depressed patients, patients with a baseline severity ≥ 14 on the HAM-D-17 had 7.23 points (95% CI 5.31-9.14 p<0.001) more improvement on the MADRS than patients meeting the exclusion criterion of ‘not meeting minimum severity threshold’. The exclusion criterion ‘not meeting a minimum severity threshold’ had no effect on the proportion of responders, but decreased the proportion that reached remission (OR 0.53, CI 0.33-0.84, p=0.01). For the subsample of psychotherapy patients, the joint analysis of exclusion criteria showed no associations with the exclusion criterion ‘not meeting minimum severity threshold’.

**Table 4:** Effects of the exclusion criteria on treatment outcome in ROM patients suffering from MDD

	**Definition of outcome**	**Influence on outcome in all patients**	**Influence on outcome in all patients adjusted**^**1**^	**Influence on outcome in psychotherapy patients**	**Influence on outcome in psychotherapy patients adjusted**^**1**^
**Current or past abuse or dependence on alcohol**					
	ΔMADRS	B = 2.77	B=2.09	-	-
		95% CI −0.85-6.39	95% CI: -1.50 -5.68		
		p=0.13	p=0.25		
	Proportion of responders	OR = 2.12	OR 2.36	-	-
		95% CI 0.80-5.63	95% CI 0.80-7.03		
		p=0.13	p=0.13		
	Proportion of remitters	OR = 1.95	OR 2.10	-	-
		95% CI 0.67-5.68	95% CI 0.61-7.27		
		p=0.22	p=0.24		
**Current or past abuse or dependence on drugs**					
	ΔMADRS	B = − 0.17	B= −1.12	B=3.37	B=3.50
		95% CI −5.35-5.02	95% CI: -6.04 -3.79	95% CI −1.04 – 7.78	95%CI −8.51-7.85
		p=0.95	p=0.65	p=0.13	p=0.11
	Proportion of responders	OR = 0.73	OR 0.70	OR 1.72	OR 1.52
		95% CI 0.24-2.22	95% CI 0.22-2.15	95% CI 0.17-17.40	95% CI 0.13-17.78
		p=0.58	p=0.53	p=0.65	p=0.74
	Proportion of remitters	OR = 0.72	OR 0.77	OR 1.19	OR 0.88
		95%CI 0.22-2.34	95% CI 0.23-2.54	95%CI 0.12-12.08	95% CI 0.07-10.39
		p=0.59	p=0.66	p=0.88	p=0.92
**Not meeting a minimum baseline severity threshold**					
	ΔMADRS	B = 6.39*	B=7.23*	B=3.37	B=3.50
		**95%CI 4.56-8.21 p<0.001**	**95%CI 5.31-9.14 p<0.001**	95% CI −1.04–7.78	95% CI −8.51-7.85
				p=0.13	p=0.11
	Proportion of responders	OR = 1.28	OR 1.53	OR 0.90	OR 0.87
		95% CI 0.83-2.00	95% CI 0.94-2.47	95% CI 0.33-2.45	95% CI 0.31-2.50
		p=0.27	p=0.09	p=0.84	p=0.80
	Proportion of remitters	OR = 0.46*	OR 0.53*	OR 0.53	OR 0.49
		**95%CI 0.30-0.70**	**95% CI 0.33-0.84**	95% CI 0.19-1.49	95% CI 0.17-1.45,
		**p<0.001**	**p=0.01**	p=0.23	p=0.20
**Previous use of medication or ECT**					
	ΔMADRS	B = 1.26	B=1.19	B=7.62*	B=5.49
		95%CI −0.42-2.95	95%CI −0.45 – 2.83 p=0.15	**95%CI 1.94-13.30**	95% CI −0.67-11.65
		p=0.14		**p<0.01**	p=0.08
	Proportion of responders	OR = 1.47	OR 1.39	OR 6.75	OR 5.46
		95% CI 1.0-2.17	95% CI 0.93-2.08	95% CI 0.82-55.83	95% CI 0.61-48.68
		p=0.05	p=0.11	p=0.08	p=0.13
	Proportion of remitters	OR = 1.53	OR 1.37	OR 4.57	OR 4.04
		95% CI 1.00-2.34	95% CI 0.88-2.13	95% CI 0.55-38.01	95% CI 0.44-37.26
		p=0.05	p=0.16	p=0.16	p=0.22

Caption:

* = exclusion of patients who meet this criterion contributes significantly to treatment outcome.

^1^ Adjusted= adjusted for age, gender and treatment modality (only in all MDD patients) and for all other exclusion criteria in the model.

B= regression coefficient: amount of additional improvement on the MADRS when patients who meet this exclusion criterion are excluded.

OR= odds ratio, the chance of response or remission when patients who meet this exclusion criterion are excluded in relation to the chance of response or remission when these patients are not excluded.

95% CI= 95% confidence interval.

For all 598 patients with MDD, exclusion of patients meeting the criterion ‘previous use of medication or ECT’ was associated with a more favourable proportion of responders and remitters in the remaining sample (OR 1.53, CI 1.00-2.34, p=0.05, unadjusted). Among the 80 psychotherapy patients, those who met the criterion ‘previous use of medication or ECT’ had 7.2 point less improvement on the MADRS than others (95% CI 1.94 -13.30, p<0.01, unadjusted). However, in the joint analysis with the other exclusion criteria, the associations were no longer significant.

The explained variance (R^2^) of the joint influence of the eligibility criteria respectively for all patients and psychotherapy patients was very small (adjusted for age, gender and type of treatment): 9 and 11% for the improvement on the MADRS; 2 and 7% for the proportion of patients who responded to therapy (50% reduction of symptoms); 4 and 7% for proportion of patients who reached remission (MADRS ≤10).

## Discussion

We evaluated the criteria for patient selection in PETs in 598 outpatients with a unipolar major depressive disorder in a Dutch general psychiatric outpatient setting. We tried to follow the model developed for the consistency of exclusion-criteria used in AETs [1,18]. However, we found a lack of consistency in the use of exclusion criteria in PETs. Only four criteria were used in at least 75% of the studies: ‘bipolar disorder or a history of a (hypo-) manic episode’; ‘schizophrenia, a history of psychosis or psychotic features’; ‘current or past abuse of or dependence on alcohol and/or drugs’ and ‘not meeting a minimum severity threshold’ (most common: cut-off score 14 on the HAM-D-17). The criterion ‘previous use of medication or ECT’, was used in 70% of the studies and would lead to exclusion of the largest percentage (44.1%) of patients from our sample. For patients receiving psychotherapy only, the largest percentage (30.8%) would be excluded because of the criterion ‘not meeting minimum severity’. In addition, we examined the influence of exclusion criteria for PETs on symptom outcome in our sample. The influence of exclusion-criteria on improvement, response and remission was small, suggesting that the most consistently used exclusion criteria are not a major threat to the generalizability of the efficacy results found in PETs.

### Comparison of exclusion criteria used in PETs to those used in AETs

To our knowledge there are no other studies on the effects of the exclusion criteria used in PETs on the generalizability to routine clinical practice. When we compared our results to those obtained in studies on the generalizability of AETs [2,18], there were some notable differences. First, PETs are less consistent in the use of exclusion criteria than AETs. The exclusion criteria ‘previous use of medication or ECT, ‘cognitive disorders’ and ‘somatic co-morbidity’ were only found in PETs. Furthermore, PETs use a lower minimum severity threshold than AETs (14 versus 18 on the HAM-D-17) and exclude cluster B personality pathology more often (57% versus 21%). However, they less often use psychiatric co-morbidity and suicide risk (resp. 24% versus 59% and 43% versus 75%) as exclusion criteria. Differences between PETs and AETs may have to do with the conduct of many AETs by pharmaceutical companies, especially for drug registration purposes. These AETs consequently have to adhere to standard exclusion criteria formulated by the authorities. Furthermore, pharmaceutical companies may want to maximize the likelihood to find an effect by selection of patients who are more severely ill. They may also minimize the risk of having their drug associated with suicide by exclusion of suicidal patients. Although not reported in PETs, this fear may also have led to patient exclusion in PETs.

### Comparison with previous research on effects of exclusion criteria on symptom outcome

We found that the exclusion of patients who are ‘not meeting the baseline severity threshold of HAM-D ≤14’ is associated with a smaller proportion of patients who reach remission (OR 0.53), while in our previous research in the same sample we found a positive association between exclusion of patients with a baseline severity of HAM-D≤17 (used in AETs) and probability of remission (OR 2.0) [7]. This finding may be explained by the fact that there were many patients in our sample who had a baseline severity between HAM-D 14 and 17 (n= 107, 18% of our study sample) who did not reach remission (78% of these 107 patients). We are currently investigating the characteristics of this specific group of patients with mild depressive symptomatology who seem to be at risk for a more chronic course of their depressive disorder. Furthermore, the treatment success in our sample was rather modest, yet in line with other research done in daily practice [51]. We commented on the differences between treatment outcome in daily practice and RCTs in previous research [52]. Interestingly, the within-group effect size of MDD treatment in our ROM population was relatively high compared to the modest remission and response percentages. An explanation for this discrepancy may be that we computed all symptom outcomes for ROM reported in Table 2, including effect sizes, on the MADRS. However, in PETs, remission and response are often measured on the MADRS or HAM-D, but effect sizes are usually computed on the BDI-II [53]. In our previous report, we investigated the effect sizes for MDD treatment on the BDI-II in our ROM population [52] and found indeed smaller effect sizes (0.85 for individual psychotherapy) than the ones based on the MADRS reported in the present study. Another explanation is that the standard deviation on the MADRS at baseline is relatively small in our ROM population, perhaps as a result of the assessment by specially trained independent research nurses.

We found that patients who used medication prior to psychotherapeutic treatment seem to benefit less from psychotherapy. Probably, these patients are non-responders or partial responders in a first treatment step for MDD and may form a more treatment resistant group. Hence, it is possible that PETs efficacy results were increased by exclusion of these patients. However, in routine clinical practice, many patients have used or are on medication before they start psychotherapy.

In line with our research on the influence of exclusion criteria of AETs on treatment outcome [7], we found an explained variance that was very small. This suggests that although many ‘real life’ patients are not eligible for RCTs on MDD [1,3,6,7], the use of eligibility criteria might not jeopardize the generalizability of the results in ‘real life’ settings. In previous research was found that patients who were eligible for AETs had a favorable treatment outcome [8], but the explained variance was not explored.

Most likely many other factors, besides eligibility, contribute to differences in outcome between RCTs and daily practice, like the Hawthorne effect [54], sociodemographic and socio-economic differences between RCT participants and ‘real life’ patients [9] and the extent of protocol adherence of both therapist and patient, in which is highly invested in RCTs and likely not to the same extent in daily practice. We elaborated more extensively on the difference between efficacy and effectiveness in a previous report [52]. Further research on factors that contribute to differences in outcome between trials and daily practice is highly recommended.

#### Strengths

We used a large sample of patients with MDD from routine outpatient clinical practice (the Leiden Routine Outcome Monitoring study [12]), for which detailed data were available, enabling analysis of a subsample of patients receiving only psychotherapy. The use of ROM data provided comprehensive data that are very representative and generalizable to ‘real life daily practice’ since there are nearly no restrictions for participation. Furthermore, we consider the fact that the Dutch healthcare system provides unrestricted access to mental healthcare as a strong quality of this research. Unrestricted access diminishes the possibility of selection bias even further.

#### Limitations

The large variability in which exclusion criteria are defined in PETs made loss of information unavoidable. In addition, in our patient sample, there was a considerable loss to follow-up of outcome measurement. However, the study sample follow-up group was similar to the lost-to-follow-up group for most sociodemographic and clinical features. Patients were lost to follow-up because they dropped out of treatment or, in 38% of the cases, remained in treatment without follow-up assessments. Loss to follow up is a problem in all studies with a more naturalistic design. For example, STAR*D reached a loss-to-follow-up of 48% in step II of the study [55].

In line with psychotherapy efficacy trials, we specifically chose to define outcome as symptom reduction or remission on an observer rated instrument in order to evaluate the generalizability of results from efficacy trials. For patients, other treatment goals might also be important, such as improvement of social functioning or quality of life. For therapists, other methods of defining treatment success, might be more useful such as clinically significant change [56]. Future effectiveness research, incorporating more definitions of outcome that are relevant to patients is therefore highly recommended. ROM can be a very useful methodology to support effectiveness research, and will also provide data to improve effectiveness research itself, as it enables a comparison between different types of treatment in daily practice, where one daily practice treatment can be a control treatment for the one under investigation. It will also provide data to explore the role of comorbid disorders in treatment and to improve diagnostic procedures in daily practice. Since there is a growing awareness that there is not just one type of major depressive disorder, in the future, ROM will hopefully be helpful in the step towards personalised MDD treatment instead of “one treatment for all”.

Another limitation of this study is the rather small size of the patient group receiving psychotherapy only. More patients received psychotherapy in combination with antidepressants, which in many cases were already prescribed by the referring physician. Unfortunately, the small number of patients with documented “current or past abuse or dependence of alcohol and/or drugs” in our psychotherapy sample prohibited exploration of this criterion. Finally, an extensive Routine Outcome Monitoring system including diagnostic instruments, symptom severity scales, both observer-rated and self report, and generic instruments measuring quality of life and social functioning is a costly investment for psychiatric practice and criticism is often heard, especially from policy makers. However, besides the opportunities to improve the quality of treatments in daily practice and the possibilities to scientifically evaluate questions that rise from daily practice, it also might be cost-effective. Since ROM provides information on treatment progress, it might enable the clinician to move to a next treatment step in case of stagnation in an earlier stage. Since ROM is relatively young, research in the field of its cost-effectiveness has, to our knowledge, not been carried out yet. It is, however, highly recommended.

## Conclusions

We found that patient selection in psychotherapy trials in MDD lacks consistency. A consistent set of exclusion criteria is recommended in order to facilitate comparison between trials and especially for daily practice to evaluate the generalizability of their results. We also found that the most consistently used exclusion criteria are not a major threat to the generalizability of results found in PETs. However, PETs do somewhat improve their results by exclusion of patients with minor depression and patients who used antidepressants prior to psychotherapy.

## Competing interests

The authors declare that have no financial or personal conflict of interest.

## Authors' contributions

All authors contributed equally to the conception and design of the study, the selection of studies, and to the final version of the manuscript. WW developed the search strategy and drafted the part of the manuscript on RCT selection and consistency in the use of exclusion criteria. RL interpreted the data and drafted the manuscript. All authors read and approved the final manuscript.

## Pre-publication history

The pre-publication history for this paper can be accessed here:

http://www.biomedcentral.com/1471-244X/12/192/prepub
